# Sodium Hydrosulfide (NaHS) Triggers Jasmonate and Reactive Oxygen Species to Boost Rice (*Oryza sativa* L.) Growth, Flowering, and Grain Yield

**DOI:** 10.3390/plants15101438

**Published:** 2026-05-08

**Authors:** Yongxing Duo, Zhigang Wu, Junfeng Dai, Yong Yang, Lisha Zhang

**Affiliations:** 1 State Key Laboratory of Biocatalysis and Enzyme Engineering, School of Life Sciences, Hubei University, Wuhan 430062, China; 202321107011629@stu.hubu.edu.cn (Y.D.); 20220146@hubu.edu.cn (J.D.); 2 Yunnan Key Laboratory for Rice Genetic Improvement, Food Crops Research Institute, Yunnan Academy of Agricultural Sciences, Kunming 650205, China; wuzhigangswu@163.com

**Keywords:** hydrogen sulfide (H_2_S), sodium hydrosulfide (NaHS), jasmonate (JA), reactive oxygen species (ROS), rice growth

## Abstract

Hydrogen sulfide (H_2_S) functions as a pivotal gaseous signaling molecule in plants, yet its role in promoting crop yield remains elusive. Here, we demonstrate that sodium hydrosulfide (NaHS) application, a donor of hydrogen sulfide (H_2_S), significantly accelerates growth, promotes flowering, and enhances grain yield in rice (*Oryza sativa* L.). Optimal NaHS treatment increased plant height, root length, and biomass accumulation, concomitant with elevated sucrose, starch, chlorophyll contents, and nitrate reductase activity. Integrated transcriptomic and proteomic analyses revealed that NaHS reprograms key biological pathways, including photosynthesis, carbon metabolism, lipid metabolism, the hormone signal transduction pathway, and reactive oxygen species (ROS) homeostasis. NaHS also remodels fatty acid metabolism, significantly increasing unsaturated fatty acids, linoleic acid (C18:2n6c), and α-linolenic acid (C18:3n3)—the latter serving as the direct precursor for JA biosynthesis—thereby fueling jasmonic acid (JA) biosynthesis. NaHS treatment also induced ROS accumulation while simultaneously activating antioxidant enzymes, maintaining redox homeostasis, and promoting cell proliferation in root meristems. Transmission electron microscopy revealed that NaHS enlarges peroxisomes and increases chloroplast oil body number, linking organellar dynamics to enhanced JA synthesis and ROS signaling. Collectively, our findings establish NaHS as a novel chemical regulator that coordinates JA and ROS signaling to boost rice growth, flowering, and grain yield, offering a promising strategy to improve crop productivity.

## 1. Introduction

Rice (*Oryza sativa* L.) is a cornerstone of food security, and enhancing its growth, accelerating flowering, and improving grain yield remain paramount objectives in agricultural research [1,2,3,4,5]. Conventional breeding and genetic engineering have contributed substantially to yield gains, but these approaches are time-consuming and encounter complex regulatory trade-offs. Thus, innovative chemical strategies that rapidly optimize developmental programming hold significant promise for advancing translational plant biology. Hydrogen sulfide (H_2_S) has emerged as a pivotal gaseous signaling molecule in plants, analogous to nitric oxide (NO) and carbon monoxide (CO), regulating diverse physiological processes including seed germination, root development, photosynthesis, stomatal movement, fruit maturation, leaf senescence, symbiotic nitrogen fixation, and various abiotic stresses [6,7,8,9,10,11,12,13,14]. As a cost-effective H_2_S donor, sodium hydrosulfide (NaHS) has been employed in rice studies, primarily focusing on stress mitigation, including phosphate starvation, salinity, aluminum toxicity, nickel stress, and drought [15,16,17,18,19,20,21,22]. Despite these stress-related advances, whether NaHS can trigger growth, flowering acceleration, and yield enhancement in rice remains unknown.

Jasmonic acid (JA) and reactive oxygen species (ROS) are integral components of plant developmental signaling networks. They act as pivotal hubs that integrate environmental cues with intrinsic growth programs. JA is a lipid-derived hormone synthesized via the octadecanoid pathway, and its biosynthesis initiates in chloroplasts, where phospholipase-mediated release of α-linolenic acid (C18:3n3) from galactolipids triggers a sequential oxidation cascade involving 13-lipoxygenase (13-LOX), allene oxide synthase (AOS), and allene oxide cyclase (AOC) to generate 12-oxo-phytodienoic acid (OPDA). Following transport to peroxisomes, OPDA undergoes β-oxidation and reduction catalyzed by OPDA reductase (OPR) and acyl-CoA oxidase (ACX) to yield bioactive JA, including methyl jasmonate (MeJA), JA-isoleucine (JA-Ile), and 12-hydroxy-JA (12-OH-JA) [23,24,25,26,27,28]. Once synthesized, JA orchestrates critical developmental transitions, including reproductive phase change, floral organogenesis, and seed maturation, while simultaneously modulating root architecture and carbon partitioning [29,30,31,32,33]. Emerging evidence indicates that H_2_S may modulate signal transduction by affecting membrane lipid homeostasis and metabolism [34,35]. Notably, H_2_S has been shown to induce jasmonic acid biosynthesis and signaling, suggesting a functional link between H_2_S and the JA pathway [36]. α-Linolenic acid (C18:3n3), derived from membrane lipids, serves as the direct precursor for JA biosynthesis. The β-oxidation steps of JA biosynthesis occur in peroxisomes to generate bioactive jasmonic acid. However, whether H_2_S modulates these processes, specifically lipid metabolism and peroxisomal function, to enhance JA biosynthesis and downstream signaling in rice remains largely unexplored.

Reactive oxygen species (ROS) play a dual role in plants, functioning as essential signaling molecules that mediate processes such as proliferation, differentiation, and programmed cell death, while also exhibiting potential cytotoxicity when in excess [37,38,39,40,41,42,43,44]. The signaling specificity of ROS is determined by their spatiotemporal accumulation dynamics, which are tightly controlled by nicotinamide adenine dinucleotide phosphate (NADPH) oxidases (Rbohs), peroxidases, and antioxidant enzymes [45,46,47]. H_2_S contributes to this regulatory network as an important antioxidant, directly or indirectly enhancing the activities of enzymes such as superoxide dismutase (SOD), catalase (CAT), and ascorbate peroxidase (APX) to maintain cellular redox homeostasis [48,49,50]. Beyond this regulation, H_2_S could directly modify protein cysteine residues through S-sulfhydration, potentially modulating the ROS-producing NADPH oxidases and cell cycle regulators [51,52,53,54]. Despite these insights, the link between H_2_S-mediated ROS regulation and subsequent growth promotion remains to be established.

Here, we proposed that exogenous application of NaHS promotes rice growth, flowering, and grain yield by coordinately modulating JA biosynthesis and ROS homeostasis. Through integrated physiological, transcriptomic, proteomic, lipidomic, and pharmacological approaches, we demonstrate that NaHS remodels fatty acid metabolism to fuel JA production while simultaneously fine-tuning ROS accumulation to stimulate cell proliferation. Our findings uncover a novel mechanism by which H_2_S acts as a key signaling hub orchestrating JA and ROS crosstalk, thereby optimizing rice developmental programming and offering a promising chemical strategy for sustainable agriculture.

## 2. Results and Analysis

### 2.1. NaHS Promotes Rice Flowering and Increases Yield

**Figure 1:**
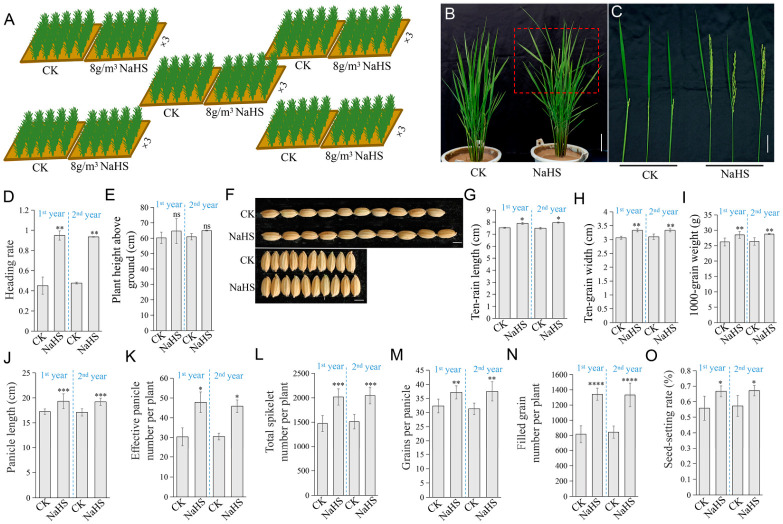
NaHS treatment promotes rice (*Oryza sativa* L.) flowering and seed size in field experiments. (**A**) Schematic diagram illustrating the grid layout of NaHS treatment in the field. (**B**,**C**) Heading phenotypes of field-grown rice under control and NaHS-treated conditions. The red dashed box highlights the rice panicles that exhibited early flowering. (**D**,**E**) Statistical analysis of heading rate (**D**) and plant height (**E**) in response to NaHS treatment across two growing seasons. (**F**) Representative images of ten-grain length and ten-grain width under control and NaHS-treated conditions. (**G**–**O**) Quantitative analysis of yield-related traits in control and NaHS-treated groups across two growing seasons, including ten-grain length (**G**), ten-grain width (**H**), thousand-grain weight (**I**), panicle length (**J**), effective panicle number per plant (**K**), total spikelet number per plant (**L**), grains per panicle (**M**), filled grain number per plant (**N**), and seed-setting rate (**O**). Data are presented as mean ± standard deviation (SD) (*n* = 10). Statistical significance was determined by Student’s *t*-test: * *p* < 0.05; ** *p* < 0.01; *** *p* < 0.001; **** *p* < 0.0001; ns indicates no significant difference. Scale bars: 7 cm (**B**), 3 cm (**C**), 0.5 cm (**F**, **top**), and 0.3 cm (**F**, **bottom**).

To investigate the effects of NaHS on rice (*Oryza sativa* L.) growth, ‘*Nipponbare*’ seedlings at a uniform developmental stage were transplanted into paddy fields. Following a one-week acclimation period, NaHS was applied to the soil at 8 g m^−2^ at three-day intervals for five consecutive treatments, after which applications ceased. This regimen was repeated across two growing seasons and plants without NaHS treatment served as the control (CK) (Figure 1A). Phenotypic parameters were assessed at key growth stages. At flowering, NaHS-treated plants exhibited significantly earlier anthesis and a higher flowering rate than the CK (Figure 1B–D). Plant height was also increased in the NaHS group, though this difference lacked statistical significance (Figure 1E). At maturity, NaHS treatment significantly improved all yield-related traits examined, including grain dimensions (ten-grain length and width), thousand-grain weight, panicle architecture (panicle length, effective panicle number, total spikelet number, and grains per panicle), and grain filling parameters (filled grain number and seed-setting rate) (Figure 1F–O). Collectively, these results demonstrate that NaHS promotes flowering and enhances grain yield in rice.

### 2.2. NaHS Promotes Rice Seedling Growth

**Figure 2:**
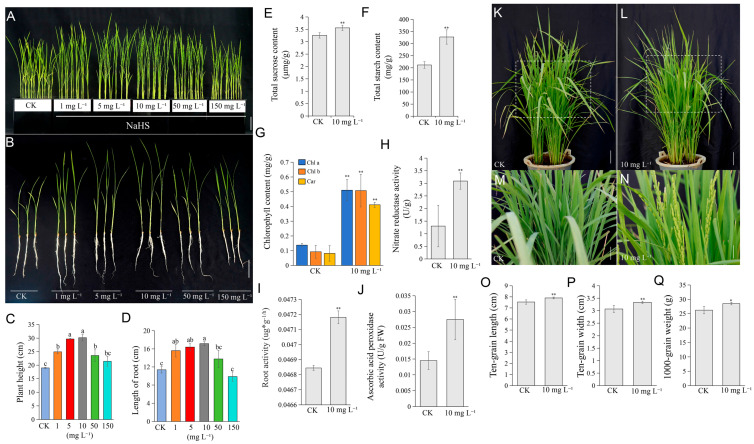
NaHS treatment enhances rice (*Oryza sativa* L.) seedling growth in hydroponic culture. (**A**–**D**) Phenotypic appearance (**A**,**B**) and quantitative data (**C**,**D**) of rice seedlings cultured with different concentrations of NaHS. (**E**–**J**) Comparative analysis of key physiological indices between the control (CK) and 10 mg L^−1^ NaHS-treated groups. The measured parameters include the contents of sucrose (**E**), starch (**F**), and chlorophyll (**G**), alongside the activities of nitrate reductase (**H**), root activity (**I**), and ascorbate peroxidase (**J**). (**K**–**N**) Panicle morphology at the heading stage of rice plants that were subjected to control or 10 mg L^−1^ NaHS treatment in hydroponic culture and subsequently transplanted to soil. The phenotype of control plants is shown in (**K**) and its magnified view (**L**), while that of NaHS-treated plants is shown in (**N**) and its magnified view (**M**). (**O**–**Q**) Comparison of ten-grain length (**O**), ten-grain width (**P**), and thousand-grain weight (**Q**) between soil-cultivated rice plants originating from hydroponic seedlings treated with control (CK) or 10 mg L^−1^ NaHS. The statistics data are means ± SD (*n* = 3). Statistical analyses in (**C**,**D**) were performed by one-way ANOVA with Tukey’s test. Different lowercase letters indicate significant differences (*p* < 0.05). Statistical analyses in (**E**–**J**,**O**–**Q**) were performed by Student’s *t*-test: * *p* < 0.05; ** *p* < 0.01. Scale bars: 3 cm (**A**), 4 cm (**B**), 7 cm (**K**,**L**), and 4 cm (**M**,**N**).

To clarify the regulatory role of NaHS in rice growth, ‘*Nipponbare*’ seedlings were hydroponically treated with five concentrations of NaHS (1, 5, 10, 50, and 150 mg L^−1^). Compared with the control (CK, 0 mg L^−1^), all NaHS treatments increased plant height to varying degrees by 31.47%, 59.76%, 64.07%, 24.1%, and 13.18%, respectively, while root length increased across all concentrations except 150 mg L^−1^ (Figure 2A–D). Based on these findings, 10 mg L^−1^ was selected as the optimal concentration for all subsequent experiments.

Compared with the CK, 10 mg L^−1^ NaHS treatment elevated sucrose and starch contents by 12.2% and 59.3%, respectively (Figure 2E,F). Additionally, chlorophyll a, chlorophyll b, and total carotenoid contents were markedly increased by 270%, 447%, and 403%, respectively (Figure 2G). Nitrate reductase (NR) activity also rose significantly (Figure 2H), as did root activity and ascorbate peroxidase (APX) activity by 0.7% and 11%, respectively (Figure 2I,J). Collectively, these results indicate that 10 mg L^−1^ NaHS coordinately enhances carbon assimilation, photosynthetic capacity, and nitrogen metabolism in rice seedlings.

Following hydroponic NaHS treatment and subsequent transplantation to soil, treated plants flowered earlier than controls (Figure 2K–N). Grain dimensions (ten-grain length and width) and thousand-grain weight were also significantly increased, consistent with field experiment results (Figure 2O–Q).

### 2.3. Transcriptome Analysis of NaHS Treatment on Rice

**Figure 3:**
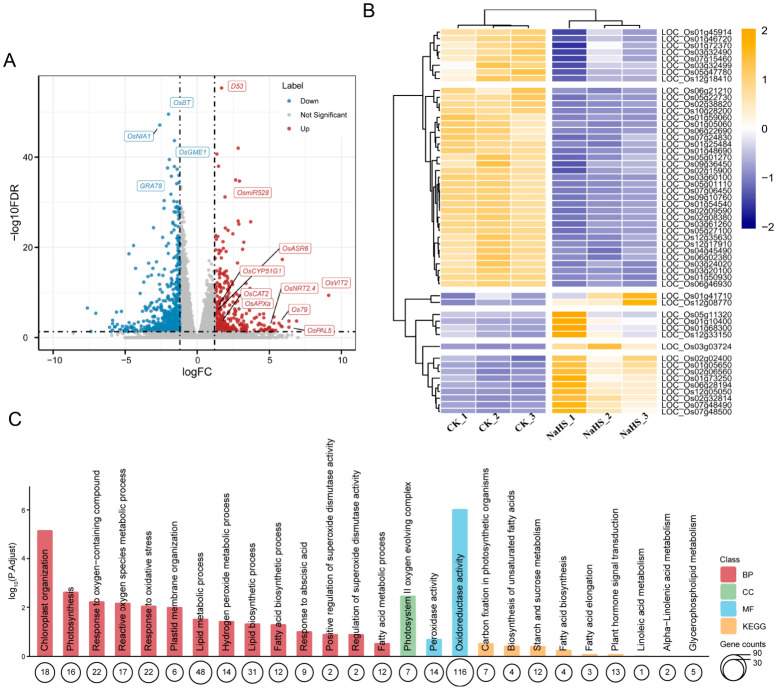
Transcriptome analysis of rice (*Oryza sativa* L.) seedlings grown hydroponically under control and 10 mg L^−1^ NaHS conditions. (**A**) Volcano plot displaying differentially expressed genes (DEGs) between 10 mg L^−1^ NaHS-treated and control (CK) rice. Significantly upregulated and downregulated genes are highlighted in red and blue, respectively, while non-significant genes are shown in gray. (**B**) Hierarchical clustering heatmap of significant DEGs across CK and NaHS-treated samples. Each row corresponds to a gene, and the color scale represents Z-score normalized expression values. (**C**) KEGG enrichment analysis of DEGs. The size of each bubble is proportional to the number of DEGs enriched in the corresponding pathway, and colors represent major functional categories.

To elucidate the molecular mechanisms underlying NaHS-mediated rice growth regulation, transcriptome sequencing was performed on CK and 10 mg L^−1^ NaHS-treated seedlings. Principal component analysis (PCA) revealed clear separation between CK and NaHS-treated samples along the PC1 axis, which explained 54.05% of the total variance (Figure S1A), indicating distinct transcriptional reprogramming upon NaHS treatment. Differential expression analysis identified 936 differentially expressed genes (DEGs), including 368 upregulated and 568 downregulated genes in response to NaHS (Figure 3A). Hierarchical clustering of DEGs revealed highly consistent expression patterns within each group and marked divergence between treatments (Figure 3B), underscoring the robustness of NaHS-induced transcriptional changes. Kyoto Encyclopedia of Genes and Genomes (KEGG) pathway enrichment analysis further demonstrated that DEGs were significantly enriched in core biological processes, including photosynthesis, the reactive oxygen species (ROS) homeostasis process, lipid metabolism and fatty acid biosynthesis, and plant hormone signal transduction (Figure 3C). Some key genes that exhibited notable expression changes included flowering regulators (*OsmiR528*, *OsGME1*, and *OsNIA1*), plant architecture determinants (*OsD53*, *OsCYP51G1*, and *OsNRT2.4*), and antioxidant defense components (*OsAPXa*, *OsCAT2*, and *OsPAL5*), implicating NaHS in the modulation of floral transition, shoot branching, and redox homeostasis (Figure S1B). In summary, these results indicate that NaHS influences rice growth and developmental processes by regulating the expression of multiple key genes associated with floral transition, plant architecture establishment, plant hormone signal transduction, and redox homeostasis.

### 2.4. Proteomic Analysis of NaHS Treatment on Rice

**Figure 4:**
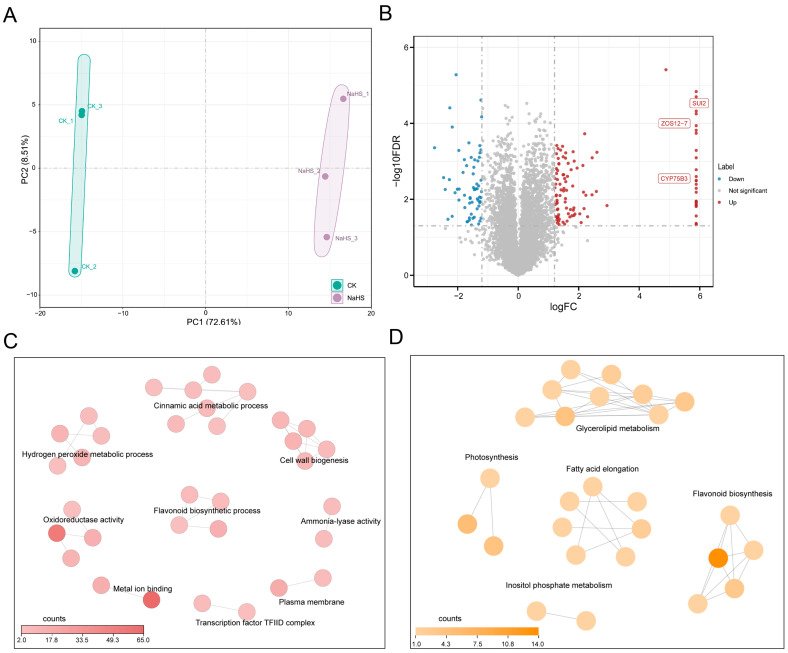
Proteome analysis of rice (*Oryza sativa* L.) seedlings grown hydroponically under control and 10 mg L^−1^ NaHS conditions. (**A**) Principal component analysis (PCA) between CK and 10 mg L^−1^ NaHS-treated rice of the proteome. (**B**) Volcano of differentially expressed proteins (DEPs) in 10 mg L^−1^ NaHS-treatment vs. CK. (**C**) Gene Ontology (GO) enrichment analysis of the identified DEPs. (**D**) KEGG pathway enrichment analysis of the identified DEPs.

Subsequently, TMT-based quantitative proteomic analysis was performed on rice seedlings treated with 10 mg L^−1^ NaHS versus the CK, quantifying 6953 proteins in total. Principal component analysis (PCA) revealed distinct separation between NaHS-treated and CK samples along the PC1 axis, which accounted for 72.61% of the total variance (Figure 4A). Differential expression analysis identified 208 significantly changed proteins (DEPs), comprising 111 upregulated and 97 downregulated proteins (Figure 4B). Gene Ontology (GO) enrichment and KEGG pathway analyses indicated that NaHS treatment coordinately enhanced flavonoid biosynthesis, the cinnamic acid metabolic process, and ammonia-lyase activity, while simultaneously maintaining ROS homeostasis via the hydrogen peroxide metabolic process and oxidoreductase activity. Additionally, lipid metabolism pathways, including glycerolipid metabolism and fatty acid elongation, were significantly enriched, providing metabolic precursors for hormone biosynthesis (Figure 4C,D).

Correlation analysis between transcriptomic and proteomic profiles revealed a modest positive correlation (R = 0.38; *p* < 2.2 × 10^−16^; Figure S2A), with a nine-quadrant plot further illustrating the synergy and divergence between mRNA and protein expression changes (Figure S2B). These results indicate that NaHS modulates rice physiology through multi-layered regulatory networks. Integrated pathway analysis revealed that NaHS significantly reshaped carbon metabolism, particularly starch and sucrose metabolism (Figure S2C).

### 2.5. NaHS Remodels Fatty Acid Metabolism and Promotes JA Synthesis

**Figure 5:**
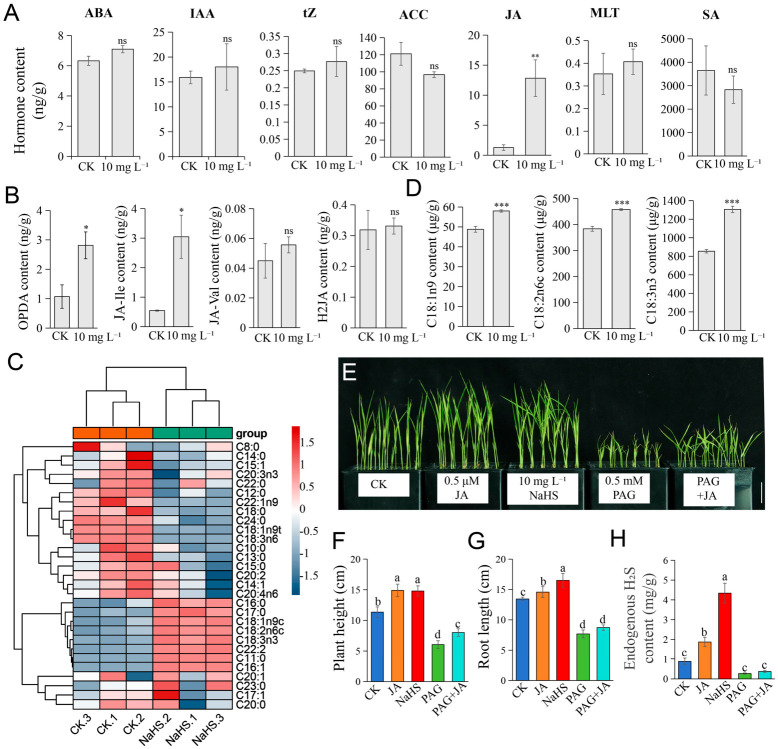
NaHS promotes rice (*Oryza sativa* L.) seedling growth by mediating JA. (**A**) Quantification of phytohormone levels in rice seedlings subjected to control (CK) and 10 mg L^−1^ NaHS treatments. The analyzed hormones include abscisic acid (ABA), indole-3-acetic acid (IAA), trans-zeatin (tZ), 1-aminocyclopropane-1-carboxylic acid (ACC), jasmonic acid (JA), melatonin (MLT), and salicylic acid (SA). (**B**) Measurement of jasmonic acid (JA) biosynthetic precursors in rice seedlings under CK and 10 mg L^−1^ NaHS treatment, including cis-(+)-12-oxophytodienoic acid (OPDA), jasmonoyl-isoleucine (JA-Ile), jasmonoyl-valine (JA-Val), and 12-hydroxyjasmonic acid (H2JA). (**C**) Hierarchical clustering heatmap showing free fatty acid content in rice seedlings subjected to control (CK) and 10 mg L^−1^ NaHS treatment. Each row corresponds to a distinct fatty acid, columns represent individual treatment groups, and the color gradient reflects relative content levels after Z-score normalization. (**D**) Comparative analysis of key unsaturated fatty acids, oleic acid (C18:1n9c), linoleic acid (C18:2n6c), and α-linolenic acid (C18:3n3), in rice seedlings under CK and 10 mg L^−1^ NaHS treatment. (**E**) Phenotypic effects of JA, NaHS, and PAG treatments on rice seedling growth. (**F**–**H**) Quantitative analyses of plant height, root length, and endogenous hydrogen sulfide (H_2_S) content, respectively, in rice seedlings subjected to the treatments outlined in (**E**). The statistics data are means ± SD (*n* = 3). Statistical analyses in (**A**,**B**,**D**) were performed by Student’s *t*-test: * *p* < 0.05; ** *p* < 0.01; *** *p* < 0.001; ns indicates no significant difference. Statistical analyses in (**F**–**H**) were performed by one-way ANOVA with Tukey’s test. Different lowercase letters indicate significant differences (*p* < 0.05). Scale bars: 4 cm (**E**).

Phytohormones play pivotal roles in plant growth and development [55]. Given the growth-promoting phenotypes observed following NaHS treatment and the enrichment of differentially expressed genes in hormone signal transduction pathways, we quantified endogenous levels of abscisic acid (ABA), indole-3-acetic acid (IAA), trans-zeatin (tZ), 1-aminocyclopropane-1-carboxylic acid (ACC), jasmonic acid (JA), melatonin (MLT), and salicylic acid (SA) using liquid chromatography-tandem mass (LC-MS/MS). Notably, NaHS treatment significantly elevated JA levels (Figure 5A), concomitant with increased accumulation of JA biosynthetic precursors, including cis-(+)-12-oxophytodienoic acid (OPDA), jasmonoyl-isoleucine (JA-Ile), jasmonoyl-valine (JA-Val), and 12-hydroxyjasmonic acid (H_2_JA) (Figure 5B).

As JA precursors are primarily derived from lipid metabolism [56], and our omics data revealed enrichment of lipid metabolism pathways among NaHS-responsive genes and proteins, we subsequently profiled fatty acid metabolites. Heatmap analysis revealed distinct fatty acid signatures between NaHS-treated and CK samples (Figure 5C), with significant increases in unsaturated fatty acids required for JA synthesis, namely C18:1n9c (oleic acid), C18:2n6c (linoleic acid), and C18:3n3 (α-linolenic acid) (Figure 5D).

To further elucidate the functional relationship between NaHS and JA, we conducted pharmacological assays using JA, NaHS, and the H_2_S synthesis inhibitor DL-propargylglycine (PAG). Both 0.5 μM JA and 10 mg L^−1^ NaHS promoted seedling growth, whereas 0.5 mM PAG exerted inhibitory effects. Co-application of 0.5 μM JA and 0.5 mM PAG alleviated PAG-induced growth inhibition (Figure 5E–G), a finding corroborated by endogenous H_2_S measurements (Figure 5H), establishing JA as a downstream mediator of NaHS signaling in rice growth regulation.

### 2.6. NaHS Promotes ROS Accumulation in Rice

**Figure 6:**
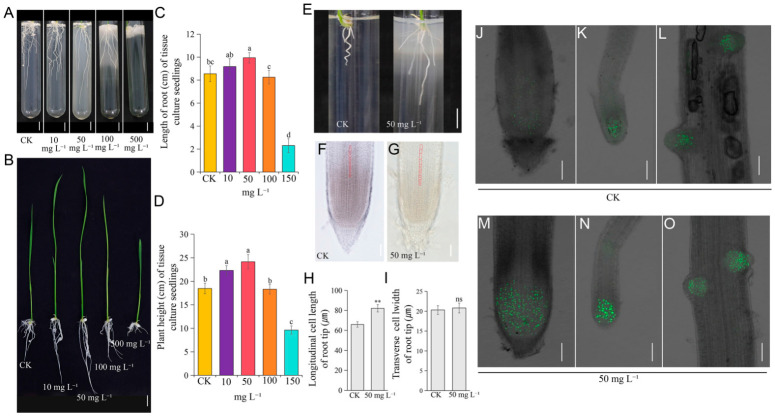
NaHS promotes rice (*Oryza sativa* L.) cell elongation. (**A**–**D**) Dose-dependent effects of NaHS on rice growth under tissue culture conditions, including representative phenotypes (**A**,**B**) and the statistics of plant height (**C**) and root length (**D**). (**E**–**I**) Cellular morphological analysis of rice root tips under CK and 50 mg L^−1^ NaHS treatment, showing root phenotype (**E**), cleared root tip cells (**F**,**G**), and the statistics of longitudinal cell length (**H**) and transverse cell width (**I**). (**J**–**O**) Assessment of cell division activity via EdU staining in primary root tips (**J**,**M**), root hairs (**K**,**N**), and root primordia (**L**,**O**) under CK and 50 mg L^−1^ NaHS treatment. The statistics data are means ± SD (*n* = 3). Statistical analyses in (**C**,**D**) were performed by one-way ANOVA with Tukey’s test. Different lowercase letters indicate significant differences (*p* < 0.05). Statistical analyses in (**H**,**I**) were performed by Student’s *t*-test: ** *p* < 0.01; ns indicates no significant difference. Scale bars: 1 cm (**A**), 2 cm (**B**), 1 cm (**E**), 100 μm (**F**,**G**), and 70 μm (**J**–**O**).

Additionally, omics analysis indicated that NaHS modulates reactive oxygen species (ROS) homeostasis. To directly assess ROS status in NaHS-treated leaves and roots, we established a tissue culture system. ‘*Nipponbare*’ rice seedlings were cultured with four NaHS concentrations (10, 50, 100, and 500 mg L^−1^), revealing dose-dependent effects on growth (Figure 6A–E). Based on these results, 50 mg L^−1^ was selected as the optimal concentration for subsequent analyses. Root tip cell morphology analysis revealed that NaHS treatment significantly increased longitudinal cell length without affecting cell width (Figure 6F–I). EdU staining to assess cell division activity further demonstrated enhanced cell proliferation signals in primary root tips, lateral roots, and emerging lateral roots following NaHS treatment (Figure 6J–O).

**Figure 7:**
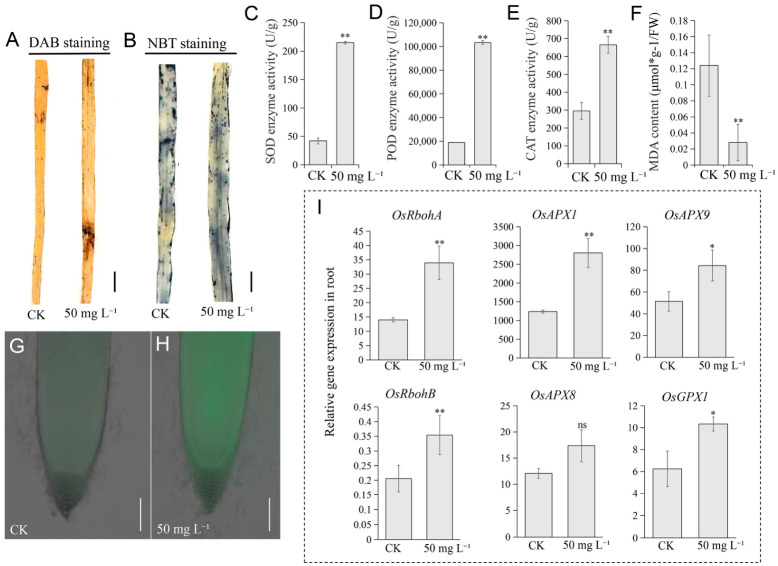
NaHS-induced reactive oxygen species (ROS) in rice (*Oryza sativa* L.) seedlings. (**A**,**B**) NBT staining (**A**) and DAB staining (**B**) of the rice leaves under CK and 50 mg L^−1^ NaHS treatment in tissue culture. (**C**–**F**) Activities of superoxide dismutase (SOD) (**C**), peroxidase (POD) (**D**), catalase (CAT) (**E**), and malondialdehyde (MDA) content (**F**) in rice seedlings treated with CK and 50 mg L^−1^ NaHS under tissue culture conditions. (**G**,**H**) ROS fluorescence signals in root tips of rice seedlings under CK (**G**) and 50 mg L^−1^ NaHS (**H**) treatment. (**I**) Relative expression levels of *OsRbohA*, *OsRbohB*, *OsAPX1*, *OsAPX8*, *OsAPX9*, and *OsGPX1* in response to NaHS treatment. The statistics data are means ± SD (*n* = 3). Statistical analyses in (**C**–**F**,**I**) were performed by Student’s *t*-test: * *p* < 0.05; ** *p* < 0.01; ns indicates no significant difference. Scale bars: 1 cm (**A**,**B**) and 0.5 mm (**G**,**H**).

Furthermore, NBT and DAB staining showed deeper intensity in NaHS-treated leaves compared to the CK, indicating elevated O_2_^−^ and H_2_O_2_ accumulation (Figure 7A,B). NaHS application also stimulated the antioxidant enzyme system, with significantly higher SOD, POD, and CAT activities relative to the CK (Figure 7C–E). Notably, oxidative damage marker MDA content was reduced despite increased ROS levels (Figure 7F). ROS fluorescence detection confirmed stronger signals in root tips of the 50 mg·L^−1^ NaHS treatment group (Figure 7G,H). RT-qPCR analysis revealed significant upregulation of ROS metabolism-related genes *OsRbohA* and *OsRbohB* in roots, alongside ROS-scavenging enzymes including *OsAPX1*, *OsAPX9*, and *OsGPX1*, but not *OsAPX8* (Figure 7I). Collectively, these results demonstrate that NaHS induces ROS accumulation while simultaneously activating antioxidant defense and cell proliferation, establishing ROS as a signaling molecule mediating NaHS-promoted growth.

### 2.7. Effects of NaHS on Peroxisomes and Chloroplasts

**Figure 8:**
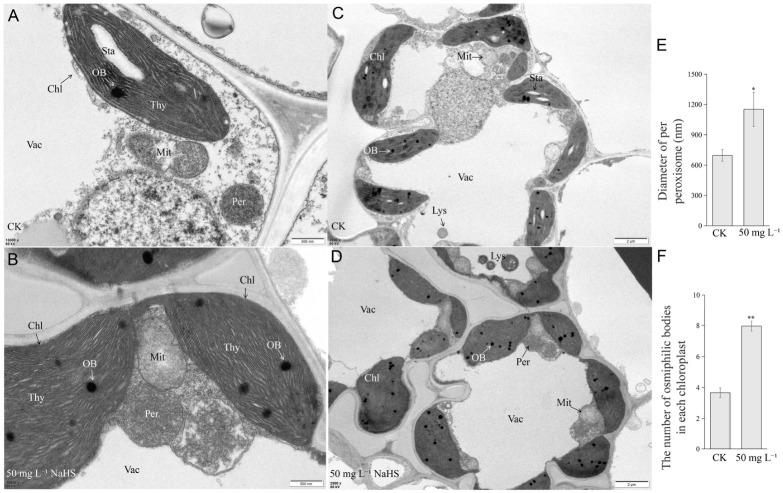
Microscopic observation of rice leaf under CK and NaHS-treatment. (**A**–**D**) Transmission electron microscopy (TEM) observation of rice leaf. Per: peroxisomes; Chl: chloroplasts; Thy: thylakoid; Vac: vacuole; OB: osmiphilic bodies; Lys: lysosome; Mit: mitochondrion; Sta: starch grain. (**E**) Statistical analysis of peroxisome diameter. (**F**) Statistical analysis of plastoglobule number per chloroplast. Statistical data are presented as mean ± standard deviation (*n* = 3). *p*-values were generated using Student’s *t*-test: * *p* < 0.05; ** *p* < 0.01; ns indicates no significant difference. Scale bars: 500 nm (**A**,**B**) and 2 μm (**C**,**D**).

ROS homeostasis and JA biosynthesis are both intimately linked to peroxisome function [23,42]. To investigate whether NaHS modulates peroxisomal activity, we examined leaf ultrastructure using transmission electron microscopy (TEM). In CK seedlings, peroxisomes exhibited regular morphology with uniform distribution, whereas NaHS treatment induced significant peroxisomal enlargement and swelling (Figure 8A,B,E). Additionally, oil body (OB) number in chloroplasts was markedly increased following NaHS treatment (Figure 8C,D,F), suggesting enhanced lipid storage or altered lipid metabolism in chloroplasts.

## 3. Discussion

Hydrogen sulfide (H_2_S) has emerged as a pivotal signaling molecule in plant stress responses, yet its potential to enhance crop productivity remains largely unexplored. In this study, we demonstrate that sodium hydrosulfide (NaHS) application significantly accelerates flowering and increases grain yield in rice (*Oryza sativa* L.), establishing H_2_S as a novel chemical regulator of developmental programming. Our integrated multi-omics analyses reveal that NaHS coordinates jasmonic acid (JA) biosynthesis and reactive oxygen species (ROS) homeostasis through metabolic remodeling, providing a mechanistic framework for H_2_S-mediated growth promotion in cereals.

The promotion of rice yield by NaHS application represents a significant advance beyond its established role in stress mitigation. Previous studies have documented NaHS-induced tolerance to phosphate starvation, salinity, and heavy metal toxicity, primarily through root architectural modification and antioxidant activation [57,58]. However, whether H_2_S could enhance yield under optimal growth conditions has remained elusive. Our experiments demonstrate that soil-applied NaHS accelerates anthesis by approximately one week and improves multiple yield components, including grain weight, panicle number, and seed-setting rate. This flowering acceleration is particularly agronomically significant, as it may shorten the growth cycle, synchronize flowering timing across varieties for hybrid breeding, and reduce exposure to late-season stresses. The consistency of yield enhancement across two growing seasons underscores the robustness and practical applicability of NaHS treatment in rice production systems. It is noteworthy that while NaHS treatment significantly increased plant height in hydroponic experiments, no statistically significant change in plant height was observed in the field experiment. We propose that this discrepancy may be attributed to several factors. First, controlled hydroponic conditions allow for direct and continuous root exposure to NaHS, whereas soil in the field experiment may adsorb or degrade NaHS, thereby reducing its bioavailability. Second, the buffering capacity of soil and interactions with soil microbiota may modulate the effective concentration of NaHS in the rhizosphere. Third, there may be inherent differences in root uptake efficiency between the two systems. Collectively, these factors likely contribute to the differential response of plant height to NaHS treatment under field versus hydroponic conditions.

The mechanistic basis of NaHS-promoted growth involves coordinated reprogramming of lipid metabolism to fuel JA biosynthesis. Our lipidomic analysis revealed significant accumulation of unsaturated fatty acids, specifically linoleic acid (C18:2n6c) and α-linolenic acid (C18:3n3), which serve as direct precursors for JA synthesis via the octadecanoid pathway. This metabolic remodeling is corroborated by transcriptomic and proteomic data showing enrichment of lipid metabolism, fatty acid elongation, and glycerolipid metabolism pathways. The finding that NaHS elevates not only JA but also its bioactive conjugates (JA-Ile and JA-Val) and precursors (OPDA, H_2_JA) establishes comprehensive activation of the JA signaling pathway. JA and PAG treatment experiments, wherein exogenous JA alleviated PAG-induced growth inhibition, confirmed JA as a downstream mediator of H_2_S signaling. This positions H_2_S upstream of JA in a novel signaling hierarchy that optimizes developmental timing for yield improvement. The peroxisomal enlargement observed through TEM further supports enhanced JA biosynthetic capacity, as peroxisomes host the critical β-oxidation steps converting OPDA to bioactive JA. In addition, NaHS treatment significantly induces the proliferation of oil bodies (OBs) in chloroplasts, which may broadly activate lipid metabolism-related pathways for JA synthesis. The discovery of the H_2_S–OBs–JA pathway reveals a new mechanism by which gaseous signaling molecules regulate plant growth through lipid metabolism and hormone signaling at the suborganelle level, not only deepening the understanding of H_2_S-mediated biological functions but also providing targets and strategies for improving crop yield by regulating OB formation or JA synthesis pathways.

Parallel to JA activation, NaHS induces moderate ROS accumulation that stimulates cell proliferation while maintaining redox homeostasis. The dose-dependent growth response to NaHS with optimal stimulation at low concentrations and inhibition at higher concentrations reflects the biphasic nature of ROS signaling. Our observations of elevated O_2_^−^ and H_2_O_2_ levels through staining, concurrent with increased antioxidant enzyme activities and reduced oxidative damage (MDA), indicate that NaHS establishes a beneficial oxidative level for promoting rice growth. This is mechanistically linked to coordinated upregulation of ROS-producing NADPH oxidases (*OsRbohA* and *OsRbohB*) and ROS-scavenging enzymes (*OsAPX1*, *OsAPX9*, and *OsGPX1*), creating a dynamic redox balance that drives cell cycle progression in root meristems. Notably, under 150 mg L^−1^ NaHS treatment, root growth was significantly inhibited, whereas plant height was not. This likely reflects the higher sensitivity of roots, which are in direct contact with NaHS, compared to shoots. Thus, the absence of shoot inhibition at 150 mg L^−1^ NaHS treatment does not contradict the expected dose–response pattern but rather indicates differential tissue sensitivity. It is possible that shoot inhibition may appear at even higher concentrations of NaHS.

Notably, ROS extensively interact with plant hormone networks, with synergistic JA-ROS crosstalk being particularly critical for growth regulation [59,60]. The JA and ROS pathways activated by NaHS share intimate connections with peroxisomal function and lipid metabolism, as evidenced by ultrastructural observations. The enlarged peroxisomes may accommodate enhanced metabolic flux for both JA synthesis and ROS detoxification, while increased chloroplastic oil bodies suggest altered lipid partitioning that supports fatty acid precursor availability. This organelle-level coordination aligns with transcriptomic enrichment of photosynthesis and carbon metabolism pathways, indicating that NaHS optimizes source–sink relationships to support accelerated development and yield formation. The modest correlation between transcriptomic and proteomic profiles highlights the importance of post-transcriptional regulation in H_2_S response, particularly for ROS homeostasis and metabolic enzyme activities. This multi-layered regulatory architecture likely enables precise spatiotemporal control of NaHS effects, ensuring coordinated growth promotion across developmental stages and tissues.

In conclusion, this study reveals that H_2_S triggers a coordinated signaling network wherein lipid metabolic remodeling fuels JA biosynthesis while redox modulation stimulates cell proliferation, ultimately accelerating flowering and enhancing grain yield in rice. These findings establish NaHS as a promising chemical regulator for cereal crop production, with optimal concentrations of 8 g m^−2^ under field conditions and 10 mg L^−1^ in hydroponic systems providing practical guidance for application. Based on the estimated canopy interception water volume in rice fields, the field application rate of 8 g m^−2^ corresponds to an equivalent soil solution concentration of approximately 80 mg L^−1^, which is considerably higher than the hydroponic optimum of 10 mg L^−1^. This discrepancy likely arises from: (1) substantial adsorption and degradation of NaHS in paddy soil; (2) reduced bioavailability within the complex soil matrix; (3) dilution effects from irrigation and rainfall; and (4) potential differences in root uptake efficiency between soil and hydroponic systems. Consequently, higher application rates are necessary under field conditions to achieve effective H_2_S levels in the rhizosphere.

Unlike genetic engineering approaches, NaHS treatment offers rapid, reversible, and cost-effective modulation of developmental programming without permanent genetic modification. Our findings position H_2_S signaling as a novel target for sustainable intensification of rice production systems. Future research should prioritize formulation optimization, precise application timing relative to key developmental transitions, and integration with existing crop management practices to maximize agronomic efficacy.

## 4. Materials and Methods

### 4.1. Plant Materials and Treatments

#### 4.1.1. Field Experiment

‘*Nipponbare*’ rice (*Oryza sativa* L.) seeds were surface-sterilized with 3% NaClO for 10 min, then soaked and germinated at 28 °C for 48 h. Subsequently, the seedlings were grown under uniform conditions in nutrient-rich soil. For the field trial, small plot squares were established at the four corners and the center of the field, complete with guard rows and drainage ditches. The experiment consisted of two treatments, CK (0 mg L^−1^ NaHS) and NaHS treatment (soil application of 8 g m^−2^ NaHS), with three biological replicates (plots) per treatment. Based on preliminary tests with a small range of NaHS concentrations—where phenotypic observations were conducted without detailed statistical analysis—the 8 g m^−2^ NaHS treatment was found to be relatively effective and was therefore selected for this trial. At the three-leaf stage, seedlings were transplanted into each plot, with a total of 36 plants per plot arranged in a 6 × 6 planting grid. During the treatment period, NaHS was applied to the soil surface every three days for a total of five applications. This field experiment was repeated for two growing seasons.

#### 4.1.2. Hydroponic Experiment

‘*Nipponbare*’ rice seeds were surface-sterilized and germinated, and uniform seedlings were selected and transferred to a growth chamber conditioned at 28/25 °C (day/night) with a 14/10 h light/dark photoperiod, a light intensity of 200 μmol m^−2^ s^−1^, and 65% relative humidity. Five-day-old seedlings were subjected to hydroponic treatment with six concentrations of NaHS—0 (CK), 1, 5, 10, 50, and 150 mg L^−1^ NaHS—each with three biological replicates. The nutrient solution was refreshed every three days. After 15 days of treatment, various physiological parameters were measured. Based on the results of NaHS treatment, a subsequent experiment was conducted using 10 mg L^−1^ NaHS (NaHS treatment) and CK (0 mg L^−1^ NaHS) for further analyses. Seedlings pre-treated with CK or 10 mg L^−1^ NaHS in hydroponic culture were transplanted into soil-filled pots with three biological replicates. For the seedling experiment, treatments with jasmonic acid (JA, purity ≥ 97%; Sigma-Aldrich, Saint Louis, MO, USA) and DL-propargylglycine (PAG, purity ≥ 98%; Sigma-Aldrich, Saint Louis, MO, USA) were performed at concentrations of 0.5 μM and 0.5 mM, respectively. All other experimental procedures, including plant growth conditions and sample handling, were as described above.

#### 4.1.3. Tissue Culture Experiment

‘*Nipponbare*’ rice seeds were surface-sterilized and aseptically placed on half-strength Murashige–Skoog (MS) medium (Coolaber, Beijing, China) [61] supplemented with 0 (CK), 10, 50, 100, and 500 mg L^−1^ NaHS, with three biological replicates per treatment. Cultures were maintained at 28 °C under light for 15 days. Based on the results, 50 mg L^−1^ NaHS was identified as the optimal concentration and used in subsequent experiments.

### 4.2. Statistical Analysis of Agronomic Traits

After NaHS treatment, comprehensive phenotyping of key agronomic traits was performed. The evaluated traits included heading rate, plant height, ten-grain length, ten-grain width, thousand-grain weight, panicle length, effective panicle number per plant, total spikelet number per plant, grains per panicle, filled grain number per plant, and seed-setting rate. Each trait was assessed using ten independent plants. Whole-plant, panicle, grain, and seedling photographs were captured under standardized conditions using a Canon 5D Mark III camera (Canon, Tokyo, Japan).

### 4.3. Sucrose Content Determination

Sucrose content was measured using a Sucrose Assay Kit (Jiancheng Bioengineering Institute, Nanjing, China). Briefly, 0.1 g fresh leaf tissue was homogenized using a mortar and pestle pre-cooled with liquid nitrogen in ice-cold 0.1 M phosphate-buffered saline (pH 7.4) to prepare a 10% (*w*/*v*) homogenate. Three biological replicates were analyzed. After centrifugation at 3500× *g* for 15 min using a Thermo Scientific Sorvall ST8 centrifuge (Thermo Fisher Scientific, Waltham, MA, USA), the supernatant was collected. Following the manufacturer’s instructions, blank, standard, and test tubes were prepared, incubated in a boiling water bath for 8 min, and then rapidly cooled. Absorbance was measured at 290 nm using a SHIMADZU UV-2600 (Shimadzu Corporation, Kyoto, Japan) spectrophotometer with ddH_2_O as the blank. Sucrose concentration (µmol g^−1^ tissue) was calculated based on absorbance differences relative to the standard.

### 4.4. Starch Content Determination

Starch content was quantified using a Plant Starch Content Assay Kit (Jiancheng Bioengineering Institute). Briefly, 0.1 g fresh leaf tissue was homogenized using a high-speed homogenizer at 13,800× *g* for 2 min in Reagent I and soluble sugars were removed by extraction at 80 °C followed by centrifugation at 4000× *g* for 10 min. The resulting pellet was gelatinized with distilled water at 95 °C for 15 min, cooled, hydrolyzed with Reagent II at 95 °C, diluted, and centrifuged. The supernatant was collected for analysis. Three biological replicates were analyzed. Blank, standard, and test tubes were prepared, incubated at 95 °C for 10 min, and cooled, and the absorbance of the supernatant was measured at 620 nm using a spectrophotometer SHIMADZU UV-2600 (Shimadzu, Kyoto, Japan) with ddH_2_O as the blank. Starch content (mg g^−1^) was calculated using the anthrone colorimetric method based on glucose equivalents derived from acid-hydrolyzed starch.

### 4.5. Photosynthetic Pigment Quantification

The extraction and calculation methods of chlorophyll and carotenoids were based on previous studies [62]. Briefly, 0.2 g fresh leaf tissue was finely chopped and immersed in 95% (*v*/*v*) ethanol for light-protected extraction overnight at 4 °C until the tissue was completely bleached. Three biological replicates were analyzed. The chlorophyll extract was then transferred to a 1 cm pathlength cuvette. Using 95% ethanol as a blank, the absorbance was measured at 663 nm, 646 nm, and 470 nm with a spectrophotometer SHIMADZU UV-2600 (Shimadzu, Kyoto, Japan). The concentrations of chlorophyll a (Chl a), chlorophyll b (Chl b), and total carotenoids (Car) were calculated using the following formulas, where *V* is the extract volume (mL), D is the dilution factor, and W is the sample fresh weight (g):

$$\mathrm{Chla}=\frac{\left(13.70\times A_{663}-5.76\times A_{646}\right)\times V\times D}{1000\times W},$$

$$\mathrm{Chlb}=\frac{\left(25.80\times A_{646}-7.60\times A_{663}\right)\times V\times D}{1000\times W}$$

$$\mathrm{Car}=\frac{\left(1000\times A_{470}-2.05\times \mathrm{Ca}-114.80\times \mathrm{Cb}\right)\times V\times D}{245\times 1000\times W}$$

### 4.6. Nitrate Reductase (NR) Activity Assay

Nitrate reductase activity was assayed using a Nitrate Reductase Activity Assay Kit (Solarbio, Beijing, China). Briefly, 0.1 g fresh rice leaf tissue was homogenized using a chilled mortar and pestle in extraction buffer. The homogenate was centrifuged at 4000× *g* for 10 min at 4 °C using an Eppendorf 5810R (Eppendorf, Hamburg, Germany), and the resulting supernatant was collected for analysis. The assay mixture, containing the sample supernatant, extraction buffer, Reagent I, and Reagent II, was prepared in a microcentrifuge tube and thoroughly mixed. Three biological replicates were analyzed. The initial absorbance (*A*_1_) was measured immediately at 340 nm. After incubation at 30 °C for 30 min, the absorbance (*A*_2_) was measured again at 340 nm. The change in absorbance (Δ*A* = *A*_1_ − *A*_2_) was calculated. NR activity was expressed as micromoles of nicotinamide adenine dinucleotide (NADH) consumed per gram of fresh weight (FW) per hour (μmol NADH g^−1^ FW h^−1^). One unit (U) of NR activity was defined as the amount of enzyme required to consume 1 μmol of NADH per hour under the assay condition.

$$\mathrm{NR}\;\mathrm{activity}=\frac{\Delta A\times V_{\mathrm{total}}}{\varepsilon \times d}\times \frac{10^{6}}{W}\times \frac{1}{T}=5.359\times \frac{\Delta A}{W}$$

where ΔA = absorbance change, ε = molar extinction coefficient of NADH (6.22 mM^−1^·cm^−1^), *d* = light path (cm), $V_{\mathrm{total}}$ = total reaction volume (mL), W = sample weight (g), and T = reaction time (h).

### 4.7. Root Activity Determination

Root activity was assessed using the triphenyl tetrazolium chloride (TTC) reduction method [63]. Briefly, 0.5 g fresh root segments from hydroponically grown seedlings was immersed in a mixture of 0.4% TTC (purity ≥ 98%; Solarbio, Beijing, China) solution and 0.067 M phosphate buffer (pH 7.8), and subjected to vacuum infiltration. After incubation in the dark at 37 °C for 2 h, the reaction was terminated by adding 1 M H_2_SO_4_. The roots were then transferred to methanol and decolorized at 30 °C until complete extraction of triphenyl formazan (TTF). Three biological replicates were analyzed. The absorbance of the TTF extract was measured at 485 nm using a spectrophotometer SHIMADZU UV-2600 (Shimadzu, Kyoto, Japan), with methanol as the blank. Root activity was calculated according to the following formula:

$$\mathrm{Root}\;\mathrm{activity}\;(\mu g\;\mathrm{TTF}\;g^{-1}\;h^{-1})=(C\times m)/(W\times h)$$

where *C* is the amount of TTF (μg) derived from a standard curve, *m* is the dilution factor of the extract, *W* is the root fresh weight (g), and *h* is the incubation time (h).

### 4.8. Ascorbate Peroxidase (APX) Activity Determination

APX activity was assayed using the Ascorbate Peroxidase Assay Kit (Solarbio, Beijing, China). Briefly, 0.1 g fresh leaf samples was homogenized on ice using a mortar and pestle in the provided extraction buffer. The homogenate was centrifuged at 12,000× *g* for 10 min at 4 °C, and the supernatant was collected as the enzyme extract. For the assay, the reaction mixture was prepared according to the manufacturer’s instructions, and the absorbance at 240 nm was measured at 5 s and 65 s using a spectrophotometer. APX activity was calculated and expressed as µmol ascorbic acid (AsA) oxidized min^−1^ g^−1^ FW. Three biological replicates were analyzed.

### 4.9. Transcriptome Sequencing

Total RNA was extracted from rice seedlings under CK and 10 mg L^−1^ NaHS treatment using TRIzol reagent, followed by DNase I (TaKaRa, Kusatsu, Shiga, Japan) treatment to remove genomic DNA. RNA integrity was assessed by 1% agarose gel electrophoresis and an Agilent 2100 Bioanalyzer (Agilent Technologies, Santa Clara, CA, USA). RNA concentration was quantified using a Thermo Scientific NanoDrop 2000 spectrophotometer (Thermo Fisher Scientific, Waltham, MA, USA). Sequencing libraries were constructed from 1 μg total RNA per sample using the Illumina TruSeq™ RNA Library Prep Kit (Illumina, San Diego, CA, USA) and paired-end sequencing [150 base pair (bp) reads] was performed on an Illumina NovaSeq 6000 platform (Majorbio Technology, Shanghai, China). Raw reads were quality-filtered using Fastp (https://github.com/OpenGene/fastp, accessed on 22 March 2026), and clean reads were aligned to the rice reference genome (IRGSP-1.0) using Hisat2 (https://daehwankimlab.github.io/hisat2/, accessed on 22 March 2026). Gene expression levels were quantified using FeatureCounts (http://subread.sourceforge.net/, accessed on 22 March 2026). Differentially expressed genes (DEGs) were identified using DESeq2 (v1.38.3, https://bioconductor.org/packages/DESeq2/, accessed on 22 March 2026) with thresholds of |log_2_(fold change)| ≥ 1 and a false discovery rate (FDR) < 0.05. Functional enrichment analysis of DEGs was performed via hypergeometric testing for Kyoto Encyclopedia of Genes and Genomes (KEGG) pathway enrichment (www.kegg.jp) and Gene Ontology (GO) term enrichment (http://geneontology.org/).

### 4.10. Proteome Sequencing

Total protein was extracted from rice seedlings under CK and 10 mg L^−1^ NaHS treatment using SDT lysis buffer (4% SDS; 100 mM Tris-HCl; pH 7.6) and quantified using a Pierce BCA Protein Assay Kit (Thermo Fisher Scientific, Waltham, MA, USA). Proteins were alkylated, digested with trypsin, and labeled using Tandem Mass Tag (TMT) (Thermo Fisher Scientific, Waltham, MA, USA) reagents. Equal amounts of TMT-labeled peptides from all samples were pooled and fractionated by liquid chromatography using a C18 column (Majorbio Technology, Shanghai, China), followed by analysis with data-dependent acquisition (DDA) on a Thermo Scientific Q Exactive HF-X mass spectrometer (Thermo Fisher Scientific, Waltham, MA, USA). Raw MS/MS data were processed using Proteome Discoverer™ software (v2.4, Thermo Fisher Scientific, Waltham, MA, USA) and searched against the UniProt *Oryza sativa* subsp. *japonica* database. Protein identification required a false discovery rate (FDR) ≤ 1%. Differentially expressed proteins (DEPs) were defined by |Fold Change| ≥ 1.5 and a *p*-value < 0.05. Functional enrichment analysis of DEPs was performed using Goatools for Gene Ontology (GO) (https://github.com/tanghaibao/goatools, accessed on 22 March 2026) and Majorbio’s proprietary KEGG pathway analysis tool (https://www.majorbio.com/, accessed on 22 March 2026) for Kyoto Encyclopedia of Genes and Genomes (KEGG) pathway enrichment on the Majorbio Cloud Platform (Majorbio Bio-pharm Biotechnology, Shanghai, China). Terms with an adjusted *p*-value < 0.05 were considered significantly enriched.

### 4.11. Determination of Hormone Content

To measure abscisic acid (ABA), indole-3-acetic acid (IAA), trans-zeatin (tZ), 1-aminocyclopropane-1-carboxylic acid (ACC), jasmonic acid (JA), melatonin (MLT), and salicylic acid (SA) content of seedlings under CK and 10 mg L^−1^ NaHS treatment, 50 mg of rice leaves was ground to powder using a ball mill (MM 400, Retsch, Haan, Germany) at 30 Hz for 3 min, then mixed with methanol/water/formic acid (15:4:1, *v*/*v*/*v*). After vortexing and centrifuging at 12,000× *g* for 10 min at 4 °C, the supernatant was dried under nitrogen, reconstituted in 100 μL 80% methanol, filtered and analyzed by ultra-performance liquid chromatography–electrospray ionization–tandem mass spectrometry (UPLC–ESI–MS/MS) using an ExionLCTM AD (Thermo Fisher Scientific, Waltham, MA, USA) coupled with QTRAP^®^ 6500+ (SCIEX, Redwood City, CA, USA) coupled with an ACQUITY UPLC HSS T3 C18 column (1.8 µm, 2.1 × 100 mm; Waters Corporation, Milford, MA, USA). The hormone content in the samples was quantified using hormone standards and data were processed using Analyst 1.6.3 (SCIEX, Redwood City, CA, USA) and MultiQuant 3.0.3 (SCIEX, Redwood City, CA, USA) [64,65]. Three biological replicates were analyzed.

### 4.12. Fatty Acid Determination

To identify differences in fatty acid content between CK and NaHS-treated seedlings, 1 g leaves was accurately weighed, ground to powder under liquid nitrogen using a mortar and pestle, and mixed with pyrogallic acid (as an antioxidant) and hydrochloric acid solution. The mixture was hydrolyzed in an 80 °C water bath. After hydrolysis, 95% ethanol was added, and the solution was extracted three times with petroleum ether/ethyl ether (1:1, *v*/*v*) solvent by vortexing. The combined supernatants were concentrated to near dryness at 40 °C. Subsequent saponification and methylation were performed as follows: the extract was refluxed with 2% sodium hydroxide in methanol at 80 °C until oil droplets disappeared, followed by the addition of 15% boron trifluoride in methanol and continued reflux. After cooling, fatty acid methyl esters (FAMEs) were extracted by shaking with n-heptane, and saturated sodium chloride solution was added to facilitate phase separation. The extraction was repeated once, and the combined n-heptane phases were evaporated to dryness under nitrogen, reconstituted in n-heptane, filtered, and subjected to gas chromatography (GC) system (Agilent Technologies, Santa Clara, CA, USA) [66]. Three biological replicates were analyzed.

### 4.13. Endogenous H_2_S Content Determination

To determine the endogenous H_2_S content in seedlings under JA, NaHS, and PAG treatments [67], 0.2 g leaves was ground into a fine powder using a mortar and pestle pre-cooled with liquid nitrogen and homogenized with ice-cold 50 mM potassium phosphate buffer (pH 6.8) containing 0.1 mM EDTA and 0.2% ascorbic acid. The homogenate was centrifuged at 12,000× *g* for 10 min at 4 °C, and the supernatant was collected. An aliquot of the supernatant was mixed sequentially with 1% zinc acetate, 20 mM N,N-dimethyl-p-phenylenediamine (DPD), and 30 mM ferric chloride (FeCl_3_). The mixture was vortexed and incubated in the dark at room temperature for 30 min. The resulting reaction product, methylene blue, exhibits a characteristic absorbance peak at 670 nm. A standard curve was generated using Na_2_S as the standard, and the endogenous H_2_S content in the samples was calculated based on the absorbance values. The absorbance was measured using a spectrophotometer SHIMADZU UV-2600 (Shimadzu, Kyoto, Japan). Three biological replicates were analyzed.

### 4.14. Root Clearing

For root tip observation, primary root tips excised from CK and NaHS-treated seedlings were immersed in a clearing solution composed of chloral hydrate, water, and glycerol (8:3:1, *w*/*v*/*v*) for 12 h [68]. Following clearing, the root tips were mounted onto glass slides with a few drops of the clearing solution, gently flattened with a coverslip, and examined under an Olympus BX53 microscope (Olympus Corporation, Tokyo, Japan).

### 4.15. EdU Staining for Cell Proliferation Analysis

To examine root apical meristem dynamics in response to NaHS treatment, cell proliferation was assessed using a YF488 Click-iT EdU Kit (Lablead Biotech, Beijing, China). The roots from CK and NaHS-treated seedlings were immersed in EdU solution and incubated in the dark for 4 h. Samples were then fixed in 4% paraformaldehyde containing Triton X-100. After three washes with phosphate buffer, the roots were incubated in 50 μL of Click-iT reaction cocktail in the dark. Following additional phosphate buffer washes, the stained samples were imaged using a Zeiss LSM710 confocal microscope (Carl Zeiss AG, Oberkochen, Germany) with excitation at 488 nm and emission detected at 495 nm.

### 4.16. 3,3′-Diaminobenzidine (DAB) and Nitroblue Tetrazolium (NBT) Staining

Uniformly sized leaf segments from CK and NaHS-treated seedlings were immersed in DAB (purity ≥ 98%; Shanghai Maokang, Shanghai, China) or NBT (purity ≥ 98%; Solarbio, Beijing, China) staining solution, respectively, and subjected to vacuum infiltration for 30 min. Samples were then incubated in the dark for 12 h until color development: DAB-stained segments exhibited a deep brown coloration indicative of H_2_O_2_ accumulation, while NBT-stained segments developed deep blue formazan precipitates indicative of superoxide anion (O_2_^−^) accumulation [69]. The leaf segments were carefully rinsed five times with distilled water and blotted dry on filter paper. Decolorization was performed by incubating the segments in 95% (*v*/*v*) ethanol at 80 °C in a water bath, with the ethanol replaced every 10 min. Once all green pigments were completely removed, the segments were rinsed three times with distilled water, blotted dry, and photographed using a Canon 5D Mark III camera (Canon Inc., Tokyo, Japan).

### 4.17. Determination of SOD, POD, and CAT Activity and MDA Content

Superoxide dismutase (SOD), peroxidase (POD), and catalase (CAT) activities, as well as malondialdehyde (MDA) content, were measured using commercial assay kits (Solarbio, Beijing, China) according to the manufacturer’s microscale protocol. Briefly, 0.1 g fresh leaf tissue was homogenized using a chilled mortar and pestle in ice-cold 50 mM phosphate buffer (pH 7.4). The homogenate was centrifuged at 12,000× *g* for 10 min at 4 °C, and the supernatant was used for enzymatic assays. SOD activity was evaluated based on the inhibition of NBT reduction at 560 nm, POD activity by guaiacol oxidation at 470 nm, CAT activity by monitoring H_2_O_2_ decomposition at 240 nm, and MDA content by a thiobarbituric acid (TBA) reaction measuring absorbance at 532 nm and 600 nm. All assays were performed with three independent biological replicates.

### 4.18. ROS Detection of Root Tip

Reactive oxygen species (ROS) in rice roots were detected using the fluorescent probe 2′,7′-dichlorodihydrofluorescein diacetate (H_2_DCFDA, purity ≥ 97%; Sigma-Aldrich, Saint Louis, MO, USA) [70]. Primary root tips excised from CK and NaHS-treated seedlings were blotted dry and immersed in H_2_DCFDA working solution. After incubation in the dark at 28 °C for 3 h, the samples were rinsed three times with phosphate buffer and mounted in a phosphate buffer–glycerol solution (1:1, *v*/*v*). H_2_DCFDA-derived green fluorescence was visualized using a Zeiss LSM710 confocal microscope (Carl Zeiss AG, Oberkochen, Germany) with excitation at 488 nm and emission collected between 500 and 550 nm.

### 4.19. Real-Time Quantitative Polymerase Chain Reaction (RT-qPCR)

Total RNA was extracted from samples using the FastPure Universal Plant Total RNA Isolation Kit (Vazyme, Nanjing, China). RNA purity, concentration, and integrity were assessed using a NanoDrop 2000 spectrophotometer (Thermo Fisher Scientific, Waltham, MA, USA). Genomic DNA removal and first-strand complementary DNA (cDNA) synthesis were performed using the HiScriptII 1st Strand cDNA Synthesis Kit (Vazyme, Nanjing, China). Quantitative real-time PCR (qRT-PCR) was performed on a Thermo Scientific ISQ9001 Real-Time PCR System (Thermo Fisher Scientific, Waltham, MA, USA) using ChamQ SYBR qPCR Master Mix (Vazyme, Nanjing, China). Each reaction was performed in triplicate. The *OsACTIN1* gene served as the internal reference control. Relative gene expression levels were calculated using the 2^−ΔΔCt^ method [71]. All primer sequences are listed in Supplementary Table S1.

### 4.20. Transmission Electron Microscope Observation

Leaf samples from CK and NaHS-treated seedlings were fixed in glutaraldehyde fixative for 3 h, washed with 0.1 M phosphate buffer (pH 7.4), and then post-fixed in 1% osmium tetroxide for 2 h. After additional washes with phosphate buffer, the tissues were dehydrated through a graded ethanol series (50%, 75%, 90%, 95%, and 100%) and infiltrated overnight in a 1:1 (*v*/*v*) mixture of acetone (Sigma-Aldrich, Saint Louis, MO, USA) and Epon 812 resin (Electron Microscopy Sciences, Hatfield, PA, USA), followed by pure Epon 812 resin overnight. The samples were then polymerized at 60 °C for 48 h. Ultrathin sections (approximately 50 nm in thickness) were cut, double stained with 2% aqueous uranyl acetate and lead citrate (Ted Pella, Redding, CA, USA) for 15 min each, air-dried overnight, and examined using a Hitachi H-7500 transmission electron microscope (Hitachi, Tokyo, Japan) for ultrastructural analysis [72].

### 4.21. Statistical Analysis

Statistical significance between two groups was determined using Student’s *t*-test in Microsoft Excel (Microsoft Corporation, Redmond, WA, USA). For comparisons among multiple groups, one-way analysis of variance (ANOVA) followed by Tukey’s multiple comparisons test was performed using GraphPad Prism 9 software (GraphPad Software, Boston, MA, USA).

### 4.22. Accession Numbers

The sequence data from this article can be found in the GenBank/EMBL libraries under the following accession numbers: *OsRbohA* (LOC_Os01g53294), *OsRbohB* (LOC_Os01g25820), *OsAPX1* (LOC_Os03g17690), *OsAPX9* (LOC_Os09g36750), *OsGPX1* (LOC_Os04g46960), and *OsAPX8* (LOC_Os02g34810).

## 5. Conclusions

This study establishes hydrogen sulfide (H_2_S) as a novel chemical regulator that promotes rice (*Oryza sativa* L.) growth, accelerates flowering, and enhances grain yield through coordinated activation of JA biosynthesis and ROS signaling. Our findings demonstrate that exogenous application of sodium hydrosulfide (NaHS) remodels lipid metabolism, increasing unsaturated fatty acid levels (C18:2n6c and C18:3n3) to fuel JA production, while simultaneously inducing moderate ROS accumulation that stimulates cell proliferation without causing oxidative damage. The integration of these pathways at the organelle level, evidenced by peroxisomal enlargement and increased chloroplastic oil bodies, optimizes source–sink dynamics and developmental programming for improved productivity. From an agricultural perspective, NaHS treatment offers rapid, reversible, and cost-effective modulation of crop performance, with optimal field (8 g m^−2^) and hydroponic (10 mg L^−1^) concentrations providing practical application guidance. These findings position H_2_S signaling as a promising target for sustainable intensification of cereal production systems.

## Data Availability

The data presented in this study are available on request from the corresponding author due to restrictions related to pending patent applications and ongoing research.

## Supplementary Materials

The following supporting information can be downloaded at: https://www.mdpi.com/article/10.3390/plants15101438/s1, Figure S1: Transcriptomic analysis of rice seedlings under CK and NaHS treatment; Figure S2: Integrated transcriptomic and proteomic analysis of rice seedlings under CK and 10 mg/L NaHS treatment. Table S1: Primer sequences used in this research.

## Abbreviations

Abbreviation	Full form	Abbreviation	Full form
12-OH-JA	12-hydroxyjasmonic acid	13-LOX	13-lipoxygenase
ABA	Abscisic acid	ACC	1-Aminocyclopropane-1-carboxylic acid
ACX	Acyl-CoA oxidase	ANOVA	Analysis of variance
AOC	Allene oxide cyclase	AOS	Allene oxide synthase
APX	Ascorbate peroxidase	AsA	Ascorbic acid
bp	Base pair	Car	Carotenoids
CAT	Catalase	cDNA	Complementary DNA
Chl a	Chlorophyll a	Chl b	Chlorophyll b
CK	Control (0 mg L^−1^ NaHS)	CO	Carbon monoxide
C18:1n9c	Oleic acid	C18:2n6c	Linoleic acid
C18:3n3	α-Linolenic acid	DAB	3,3′-Diaminobenzidine
DDA	Data-dependent acquisition	DEGs	Differentially expressed genes
DEPs	Differentially expressed proteins	DPD	N,N-dimethyl-p-phenylenediamine
EDTA	Ethylenediaminetetraacetic acid	EdU	5-Ethynyl-2′-deoxyuridine
FAMEs	Fatty acid methyl esters	FDR	False discovery rate
FW	Fresh weight	GC	Gas chromatography
GO	Gene Ontology	H_2_DCFDA	2′,7′-Dichlorodihydrofluorescein diacetate
H_2_JA	12-Hydroxyjasmonic acid	H_2_S	Hydrogen sulfide
IAA	Indole-3-acetic acid	JA	Jasmonic acid
JA-Ile	Jasmonoyl-isoleucine	JA-Val	Jasmonoyl-valine
KEGG	Kyoto Encyclopedia of Genes and Genomes	LC-MS/MS	Liquid chromatography-tandem mass spectrometry
MDA	Malondialdehyde	MeJA	Methyl jasmonate
MLT	Melatonin	MS	Murashige–Skoog
NADH	Nicotinamide adenine dinucleotide (reduced form)	NADPH	Nicotinamide adenine dinucleotide phosphate (reduced form)
NaHS	Sodium hydrosulfide	NBT	Nitroblue tetrazolium
NO	Nitric oxide	NR	Nitrate reductase
Ob	Osmophilic body	OPDA	12-Oxo-phytodienoic acid
OPR	OPDA reductase	PAG	DL-propargylglycine
PCA	Principal component analysis	POD	Peroxidase
ROS	Reactive oxygen species	RT-qPCR	Real-time quantitative polymerase chain reaction
SA	Salicylic acid	SD	Standard deviation
SOD	Superoxide dismutase	tZ	trans-Zeatin
TEM	Transmission electron microscopy	TMT	Tandem mass tag
TTC	Triphenyl tetrazolium chloride	TTF	Triphenyl formazan
UPLC–ESI–MS/MS	Ultra-performance liquid chromatography–electrospray ionization–tandem mass spectrometry	UV	Ultraviolet
