# Supplementary material for: Sodium Hydrosulfide (NaHS) Triggers Jasmonate and Reactive Oxygen Species to Boost Rice (Oryza sativa L.) Growth, Flowering, and Grain Yield
Source: Plants (Basel). 2026 May 8;15(10):1438. doi: 10.3390/plants15101438 (PMC13211098; doi:10.3390/plants15101438)
Supplement: Supplementary file 1 [file plants-15-01438-s001.zip › plants-4244355-supplementary.pdf]

Supplementary figure S1

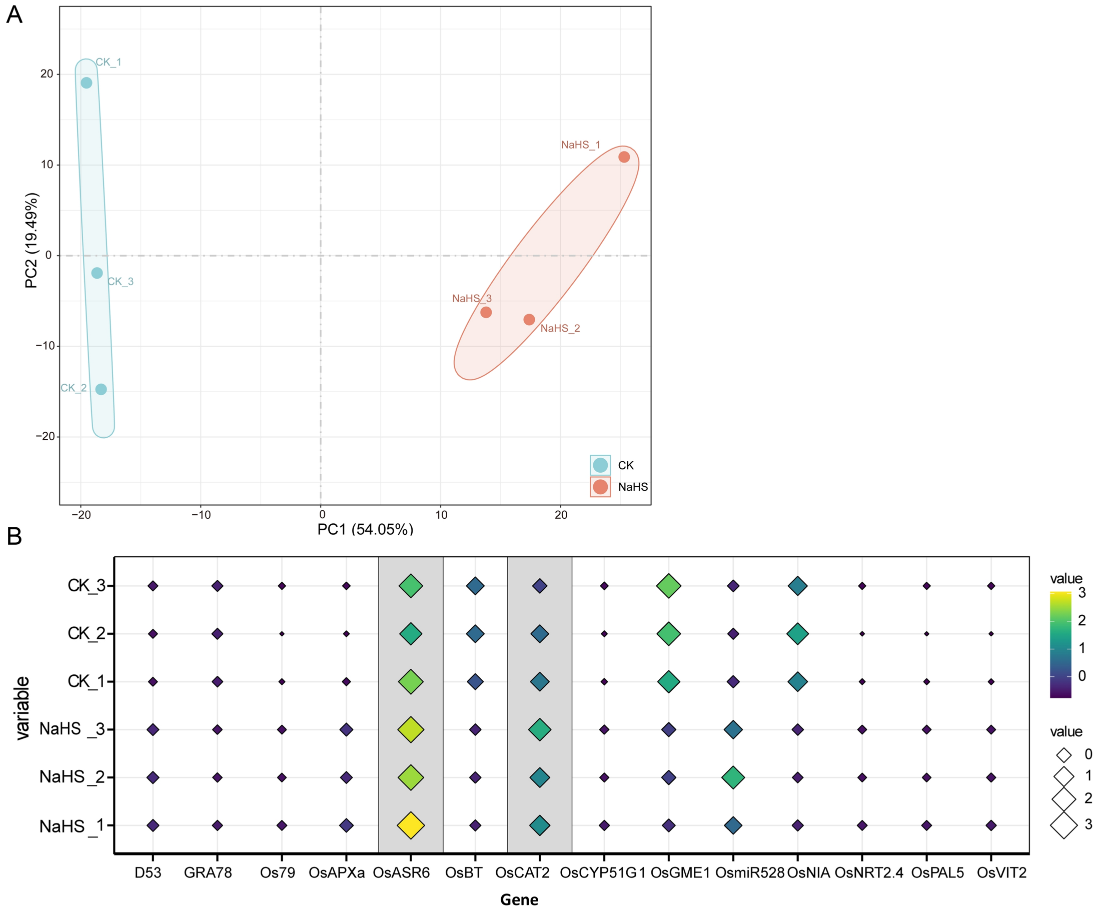

Figure S1. Transcriptomic analysis of rice seedlings under CK and NaHS treatment.

(**A**) Principal component analysis (PCA) of transcriptome profiles from rice seedlings under control (CK) and 10 mg/L NaHS treatment.

(**B**) Expression profiles of selected key genes. Dot size reflects the expression level across samples, and color intensity represents the statistical significance of expression changes.

Supplementary figure S2

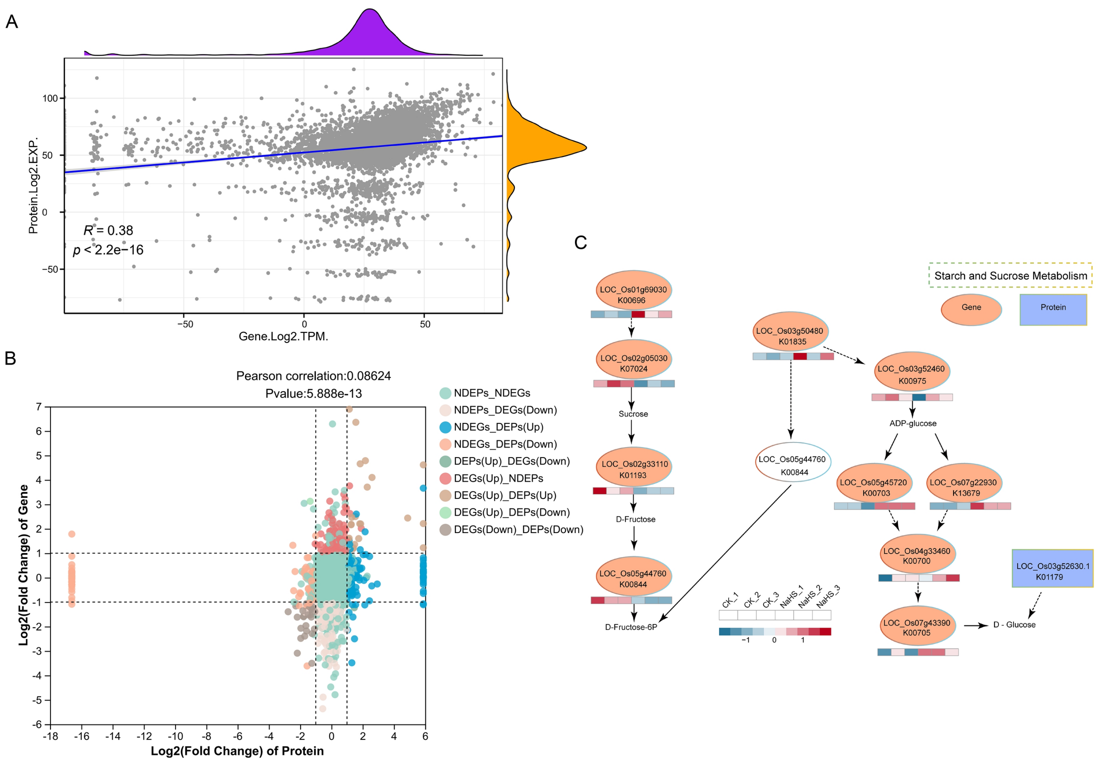

Figure S2. Integrated transcriptomic and proteomic analysis of rice seedlings under CK and 10 mg/L NaHS treatment.

(**A**) Correlation analysis between transcriptomic and proteomic datasets.

(**B**) Nine-quadrant analysis depicting the relationship between differentially expressed genes (DEGs) and differentially expressed proteins (DEPs). Quadrants are defined as follows:

1: Down-DEGs/Up-DEPs; 2: Non-DEGs/Up-DEPs; 3: Up-DEGs/Down-DEPs;

4: Up-DEGs/Non-DEPs; 5: Non-DEGs/Non-DEPs; 6: Down-DEGs/Non-DEPs;

7: Up-DEGs/Up-DEPs; 8: Non-DEGs/Down-DEPs; 9: Down-DEGs/Down-DEPs.

(**C**) Starch and sucrose metabolism pathway highlighted in the integrated transcriptomic and proteomic analysis.

Supplementary Table S1. Primer sequences used in this research.

Primer name	Primer sequences(5′–3′)
OsRbohA RT-F	TCTCATCATCTGTTCATCATTGTGTATATCG
OsRbohA RT-R	CCAGACCTGAAGAACCTTAGAATCCT
OsRbohB RT-F	GCTCACAGCAGAAGAGGTTAAGGAGAT
OsRbohB RT-R	GCTTCAGATGGTGACTGAAGCAAT
OsAPX1 RT-F	AAGGTGCCACAAGGAAAGATCTGG
OsAPX1 RT-R	ATCTGCAGCATATTTCTCGACGAGT
OsAPX8 RT-F	ATGCTTTGAAGCTTATCCAACCAATCAA
OsAPX8 RT-R	GCTGTGACATCAACTCGTCCATAT
OsAPX9 RT-F	ACCACTTAAGTTTGACAACTCTTACTTCCTAG
OsAPX9 RT-R	GTGTGATTCAGCGTAGTCCTTGAAGAA
OsGPX1 RT-F	GTTGATGTCAACGGTAACAATGCTG
OsGPX1 RT-R	GTAGTGCAGGACATTACCTCAATACTAAGA
Actin-RT-F	GACCCAGATCATGTTTGAGACCT
Actin-RT-R	CAGTGTGGCTGACACCATCAC
